# Curcumin as an Anticancer Agent in Malignant Mesothelioma: A Review

**DOI:** 10.3390/ijms21051839

**Published:** 2020-03-07

**Authors:** Alfonso Baldi, Antonio De Luca, Patrizia Maiorano, Costantino D’Angelo, Antonio Giordano

**Affiliations:** 1 Department of Environmental, Biological and Pharmaceutical Sciences and Technologies, University of Campania “L. Vanvitelli”, 81100 Caserta, Italy; 2 Department of Mental and Physical Health and Preventive Medicine, Section of Human Anatomy, University of Campania “Luigi Vanvitelli”, 80138 Naples, Italy; antonio.deluca@unicampania.it; 3 Department of Medical Biotechnologies, University of Siena, 53100 Siena, Italy; patriziamaiorano@gmail.com (P.M.); costantinodangelo92@gmail.com (C.D.); president@shro.org (A.G.); 4 Sbarro Institute for Cancer Research and Molecular Medicine, Center for Biotechnology, College of Science and Technology, Temple University, Philadelphia, PA 19019, USA

**Keywords:** curcumin, mesothelioma, piperine, phytochemical, therapy

## Abstract

Malignant mesothelioma is an infrequent tumor that initiates from the mesothelial cells lining of body cavities. The great majority of mesotheliomas originate in the pleural cavity, while the remaining cases initiate in the peritoneal cavity, in the pericardial cavity or on the tunica vaginalis. Usually, mesotheliomas grow in a diffuse pattern and tend to enclose and compress the organs in the various body cavities. Mesothelioma incidence is increasing worldwide and still today, the prognosis is very poor, with a reported median survival of approximately one year from presentation. Thus, the development of alternative and more effective therapies is currently an urgent requirement. The aim of this review article was to describe recent findings about the anti-cancer activity of curcumin and some of its derivatives on mesotheliomas. The potential clinical implications of these findings are discussed.

## 1. Malignant Mesothelioma: A Growing Health Emergency

Malignant mesothelioma (MM) is an infrequent and extremely aggressive neoplasm, which arises from the mesothelial cells of the pleura and from other serosal surfaces such as peritoneum, pericardium and tunica vaginalis, and it is primarily associated with exposure to asbestos fibers, with a latency period of 20–40 years. MM incidence is increasing worldwide, and it is estimated to peak over the next 15 years with a growth of 5.4% per year. It has been estimated that, only in Europe, this cancer will be responsible of more than 250,000 deaths in the next 30 years [1,2,3]. The commencement of MM symptoms is often insidious and not specific. Effective MM diagnosis is problematic and frequently necessitates invasive surgery as well as multiple time-consuming tests. MM is a characteristically “diffuse” tumor. In the early phase, the tumor consists of numerous slight nodules or plaques covering visceral and parietal serosal surfaces [4,5]. In the late stage of the disease, there is the fusion of the individual nodules with the formation of a diffuse sheet-like thickening that encloses and compresses the viscera with matting of the affected structures [5]. In detail, the tumor, when affecting the pleura, usually permeates the fissures between the pulmonary lobes and can extend through the hemi-diaphragms into the abdominal cavity [6]. The majority of MMs manifests as a symptom of onset with effusion [7]. Metastasis is a late event, but at death, may be extensive [1,2,3].

From the histological point of view, MMs are subtyped into three major categories: epithelioid, sarcomatoid and biphasic (mixed) [6]. Epithelioid MMs are made up of cuboidal and flattened cells forming tubular structures, with great quantities of eosinophilic cytoplasm and round nuclei with a single and evident nucleolus. Mitoses are sporadic. Furthermore, the most common growth patterns of epithelioid MMs are, in addition to tubular, tubulopapillary, papillary, trabecular, acinar, microglandular, solid, and pleomorphic. The latter consists of anaplastic cells and often, giant cells, and is associated with very poor prognosis so that classification of pleomorphic epithelioid MM as a separate new type of MM is under consideration, but for the time being, it is still classified as a subtype of epithelioid MM [6,7]. Sarcomatoid MMs are formed by spindle cells that may fluctuate from a well differentiated to an anaplastic appearance. Elements of osseous, chondroid, and leiomyomatous or rhabdomyosarcomatous metaplasia, as well as the presence of giant cells or interlacing, fibrosarcoma-like bundles are also observed. In biphasic (mixed) MM both components, epithelioid and spindle cells are observed and the transition from one to another can be sudden or gradual. The possibility of observing the mixture of the components of the tumor is also very common. Nevertheless, it is very common to detect two or more histologic patterns in the same tumor, especially if more sections are analyzed [8]. The differential diagnosis between MM and primary lung cancer invading the pleura or tumors metastasizing to pleura can be a very difficult task for the pathologist. Iimmunohistochemistry is still the most effective diagnostic tool to solve this problem. The antibodies commonly used are divided into two key categories: (a) positive markers for MM and (b) negative markers for MM. Usually, a panel of four markers (two positive and two negative) is applied for the distinction between lung adenocarcinoma and MM. The top combination comprises calretinin and cytokeratin 5/6 (or either WT1 or D2-40) as positive markers and CEA and MOC-31 (or either B72.3 or Ber-EP4) as negative markers [6,7].

Regarding the different incidences of the three major sub-types, about 50% of the cases are epithelioid, 30% are biphasic and 15–20% are sarcomatoid. The recognition of the different histologic subtypes is required for a correct diagnosis. Moreover, it is still the most important prognostic marker for MM and has importance for the operability of MM patients in centers offering surgical treatment for MM. Indeed, most centers operate only pts with epithelioid MM or biphasic MM with dominance of the epithelioid component, but not those with dominance of the sarcomatoid component or purely sarcomatoid MM. Finally, recent data have indicated that epithelioid MM and sarcomatoid MM express different levels of PD-L1 and VISTA, with different implications for immunotherapy [8].

The prognosis of MM is very poor, with a reported median survival of approximately one year from presentation [9,10]. This phenomenon is due to the long latency of the tumor (10–30 years) to late diagnosis as the tumor does not show symptoms if not at an advanced stage and to the peculiar resistance to the standard therapeutic modalities, such as surgery, chemotherapy and radiotherapy treatments [11,12]. Thus, the development of alternative and more effective therapies is currently an urgent requirement [13,14,15,16].

Due to the low incidence of the disease, only a limited number of randomized studies have been performed [3]. The stated response rates to the different therapeutic protocols fluctuate from 10% to 45% with no significant advantage in terms of time of survival, which is between 4 and 9 months [9]. Various chemotherapy agents have been verified in different combinations so far; among the most frequently utilized are doxorubicin, cyclophosphamide, cisplatin, carboplatin, gemcitabine, and pemetrexed [10]. Unfortunately, the combination of these drugs provides, in the best case, only marginal advantage over mono-therapy [3].

The role of surgery in the management of MM remains debated. Hypothetically, the whole surgical resection should be considered the most effective treatment. However, because of the usual thoracic diffusion of the disease, complete resection with negative margins is rarely obtained. Hence, the term cytoreduction has usually been employed no matter the type of resection performed to indicate removal of the vast bulk tumor with microscopic residual left behind on the visceral pleura, mediastinum, diaphragm and chest wall [3]. Certainly, surgery is incapable of completely removing a neoplasm arising from multiple sites on the parietal and visceral pleura or peritoneum. Nevertheless, surgery represents an important step within a multimodality strategy, which has recently been demonstrated to improve quality of life and survival [9,10]. Hence, the term cytoreduction has usually been employed to describe all types of resection performed to indicate removal of the vast bulk tumor with microscopic residual left behind on the visceral pleura, mediastinum, diaphragm and chest wall [3]. The magnitude of the operation is usually tailored by taking into consideration several factors, including the patient’s age, performance status, comorbidity and, finally, the surgeon’s experience.

Several studies have also shown that the development of malignant mesothelioma is related to immune suppression by HIV, thus resulting in a complication of AIDS similar to the development of other malignant neoplasms in patients with AIDS [17,18]. Nevertheless, immunotherapy, particularly immune checkpoint inhibitors, has generated much excitement because of data suggesting the potential value of immune checkpoint inhibitors for patients who have failed chemotherapy [19,20,21].

A major restriction of the conventional treatments for cancer is associated with serious side effects that chemotherapy drugs may cause and to the development of cancer resistance. Unfortunately, this is particularly true in the case of MM, which is a tumor highly refractory to standard chemotherapy treatments, surgery and radiotherapy [3,22,23]. This is why research is very active to identify and define new therapeutic protocols that are more effective and possibly less toxic for mesothelioma patients.

## 2. Phytochemicals as Anti-Cancer Molecules: The Example of Curcumin

Some advances in cancer therapies have been recently reached thanks to the introduction of alternative approaches, including the use of several phytochemicals which are able to significantly reduce tumor progression and to improve healing and survival [24,25,26].

Phytochemicals in food science include an assortment of plant components which have different structures capable of helping health [24]. Phytochemicals, known as secondary metabolites, are non-nutritive bioactive chemical composites generated by plants [25]. They are named non-nutritive since they are produced by plants only in specific cells and not by energy metabolism nor by catabolic or anabolic metabolisms. To date, 10,000 phytochemicals have been documented, including pre- and pro-biotics, polyphenols, carotenoids, steroids, and thiosulfate; nevertheless, a lot continue to be unidentified [27]. Phytochemicals are vital for plant metabolism as they keep away pests and sunlight and control plants growth.

Phytochemicals display differences in metabolism and deposition because of the unpredictability in the absorption, distribution, and excretion of phytochemical pharmacokinetics. Examples of variability of sources of phytochemicals comprise hydroxylation in the liver that cause secondary oxidation products as well as reduction, dihydroxylation and demethylation, that are performed by gut bacteria and are responsible for the formation of more biologically active metabolites [27]. Overall, the great majority of phytochemicals exist as glycosides or other conjugates in plant food; therefore, hydrolyzation is a crucial procedure for absorption and it is generally accomplished either by brush-border membrane-bound β-glucosidases or by gut bacterial β-glucosidases in the colon or lower small intestine. After absorption, the most important step is glucuronization, which makes the compounds suitable to be excreted in the bile or urine [27,28,29].

Recently, phytochemicals have arisen as modulators of critical cellular signaling pathways and health enhancement. Indeed, they confer important anti-cancer properties acting as effective tumor growth reducers with no obvious associated side effects [27]. Several studies claim that natural molecules can have anticancer properties sensitizing cancer cells to standard therapy [28,29].

In recent years much attention has been paid to the anti-cancer action of the active ingredients present in turmeric (curcuma longa), also known as “curcuma domestica”. This is a perennial herbaceous plant of the ginger family (Zingiberaceae) [30]. In detail, among the over 300 active components of this plant, research has focused on the properties of curcumin, a substance obtained from its root, characterized by a yellow or orange pigment [31]. It is the main biologically active component of this plant and constitutes the major basis for the medicinal properties of tumeric [32]. Curcumin has been widely used for centuries for the treatment of several diseases, its usage dating back more than 2500 years in Asia, especially in traditional Indian medicine (Ayurveda) [33]. It has been utilized for various health problems, including several skin diseases, wounds, sinusitis, eye infection, rheumatism, dyspepsia, stress and depression Helicobacter pylori infection, peptic ulcer and irritable bowel syndrome, and placental diseases [34,35,36,37,38,39,40,41]. Interestingly, the first scientific study on the therapeutic properties of curcumin was published in 1937 [42]. Since then, a plethora of observations have been produced on the therapeutic effects of curcumin in various pathologies and currently, several thousand scientific articles on curcumin are registered in PubMed [32].

**Figure 1:**
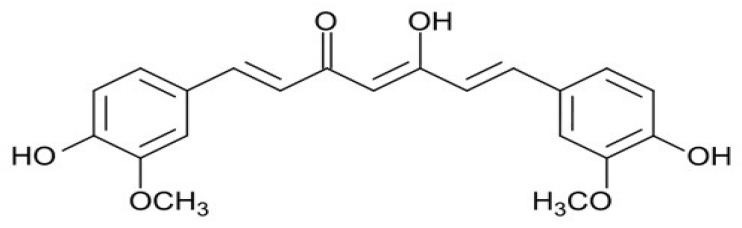
The chemical structure of curcumin.

The chemical structure of curcumin consists of two phenyl rings substituted with hydroxyl and methoxyl groups connected via a seven-carbon keto-enol linker (Figure 1). In more detail, the properties described for curcumin by acting on different kinases, cytokines, enzymes and transcription factors, include anti-inflammatory, anti-oxidant, anti-microbial, immunomodulatory, hypoglycaemic, hepatoprotective, and reno-protective effects [32]. In more detail, several molecular mechanisms have been proposed to act on different pathways. Among them, we can indicate: interaction with the arachidonic acid metabolism, inhibition of the signal transducer and activator of transcription-3 pathway, inhibition of the activities of c-Jun/AP- 1, protein kinase C, epidermal growth factor, cyclooxygenase-2, lipoxygenase, ornithine, and nuclear factor-kappa B, among others [30,31,32,33,34]. Nevertheless, other studies have demonstrated an association with some pathways regulating angiogenesis and apoptosis, such as matrix metalloproteinase, vascular endothelial growth factor, and caspase and mitochondria-dependent apoptosis [32].

Regarding the anti-cancer effects of curcumin, one of the first observations dates back to 1985, when Kuttan et al. proposed this substance as a potential chemotherapy [43]. The rationale behind these anti-cancer properties of curcumin is that inflammation is one of the factors for cancer development and this substance could influence it by its anti-inflammatory effects [44]. Numerous articles described curcumin’s antitumor activity on several different mailgnant tumors [45], showing its ability to target multiple cancer cell lines.

The anti-cancer activity of curcumin is based on its ability to block the proliferation of tumor cells, modulating cell-cycle regulatory proteins involved in the pathogenesis and the prognosis of several cancers [42]. In more detail, curcumin induces a selective cytotoxicity toward cancer cells, blocking the expression of molecules involved in cancer growth [46,47,48]. Moreover, curcumin is able to contrast the multidrug resistance of cancer cells down-regulating proteins responsible for the high drug efflux in multi-drug-resistant cancer cells [49]. Finally, curcumin has the ability to act in vivo, synergizing drug effects and thus increasing the global efficacy [50,51,52,53,54].

Curcumin is a low-cost, easily accessible and low-toxicity element; therefore, it is an ideal chemo-preventive agent. Precisely for these reasons, curcumin has been included by the US National Cancer Institute among the most promising chemo-preventive agents and considered in clinical trials since the 1980s. Despite this, the pharmacological potential of curcumin is severely restricted due to its poor water solubility, photodegradation, chemical instability and rapid metabolism as well as to its poor systemic bioavailability after oral administration [54,55,56]. In fact, the glucuronidation and sulfation of curcumin happen very soon after in vivo administration and the produced metabolites are rapidly excreted from feces, urine and bile [57]. To have an idea of the low bioavailability of curcumin, these data are very indicative: ingestion of 2 g of pure curcumin results in the presence of only 10 ng/mL curcumin in plasma [58]. Indeed, its poor bioavailability and high potential to interfere with other drugs constitute real obstacles for precisely defining the real biological and clinical anti-cancer properties of curcumin. Nevertheless, more attention should be paid to the formulations used, since in most of the cited in vivo studies and clinical trials, no-standardized curcuminoid mixtures have been used [57].

In this regard, numerous attempts aimed at increasing the efficacy and bioavailability of curcumin have been made. In particular, attention has been paid to designing suitable formulations using nanocarriers for delivery and targeting [58,59,60].

For the rational design of the nano-formulations, numerous aspects should be well thought-out in order to increase the effectiveness and improve the cellular targeting of anticancer agents. The most well studied ones include nanoparticle size and shape, surface properties, and nanoparticle targeting ligands [59]. One of the most commonly used curcumin delivery systems are the polymeric nanoparticles utilized to improve its biological activity. Biocompatible and biodegradable polymers are ideal in drug delivery systems due to their lower risk of toxicity. Therefore, biodegradable synthetic polymers such as PLGA (poly (d, l-lactic-co-glycolic acid) and natural polymers such as silk fibroin and chitosan have become widely used in drug delivery [60]. Nevertheless, nanoscale liposomes are emerging as one of the most advantageous drug delivery systems for anticancer agents [58]. A liposome consists of a phospholipid bilayer shell and an aqueous core which makes it a superlative transporter for capturing both hydrophobic and hydrophilic compounds. Numerous liposome preparations have been utilized to encapsulate curcumin. Concerning nanogels, they have gained significant consideration in recent years as a promising drug delivery system, but only a few studies have investigated curcumin-nanogel delivery in cancer therapy [59]. Finally, cyclodextrins are cyclic oligosaccharides which comprise a hydrophilic outer layer and a lipophilic core. In drug delivery, these complexes offer numerous advantageous properties, including greater solubility, amplified bioavailability, and enhanced stability of the loaded drug. However, few studies have used cyclodextrins as carriers in curcumin delivery to improve bioavailability [59].

Additional approaches have been based on the mixture of curcumin with other substances. Among them, the most promising is the co-administration of curcumin with Piperine, an alkaloid of black pepper and long pepper. Piperine significantly enhances curcumin bioavailability by up to 2000% by preventing its metabolism through the inhibition of glucuronidation processes [61].

## 3. Laboratory and Clinical Studies Carried Out on the Effects of Curcumin in Malignant Mesothelioma

In the scientific literature, there is a limited number of laboratory studies in which the antiproliferative effects of curcumin in various in vitro and in vivo models of mesothelioma were investigated. In Table 1, the most relevant studies are indicated, briefly describing the main results obtained by the research groups.

One of the first observation has been produced by Wang et al. [62]. This research group used different human and murine mesothelioma cell lines (H2373, H2452, H2461, H226 and AB12) to analyze mechanisms of curcumin-dependent growth suppression. In detail, the article demonstrated that this inhibition was dose- and time-dependent and that curcumin pre-treatment sensitized MM cells to cisplatin. From a molecular point of view, curcumin treatment was able to activate pro-apoptotic proteins, stimulating PARP cleavage and apoptosis. Finally, the growth-suppressive action of curcumin was also confirmed on murine MM cell-derived tumors in vivo in part by stimulating apoptosis.

Interestingly, in a later work, Yamauchi et al. investigated the effects of curcumin on the human derived MM cell line ACC-MESO-1 and found that the dose-dependent reduced cell viability caused by curcumin was not attributable to apoptosis but to autophagy, as also demonstrated by the detection of autophagosomes on electron microscopy [63].

In the same year, an interesting paper by Miller et al. was published that shed new light on the biological mechanisms responsible for MM cell-growth suppression caused by curcumin [64]. In detail, the authors demonstrated that in in vitro models with mouse and human MM cell lines, curcumin induced pyroptosis through the activation of caspase-1 while preventing processing of pro-inflammatory cytokines interleukin-1β and interleukin-18. In more detail, the authors proposed that curcumin was able to avoid cytokine processing by stalling priming of cytokine precursors through inhibition of the NF-kB pathway. Moreover, curcumin was able to downregulate levels of inflammosome-related genes. Consequently, the authors concluded that the growth-suppressive activity of curcumin on MM cells was through the induction of pyroptosis and protection against inflammation.

In a different approach, curcumin-loaded nanoparticles were produced, where the nanoparticles were made up of an amphiphilic blend of poloxamers and PLGA to confer them stealth properties [65]. These particular nanoparticles were able to be internalized by the MSTO-211H MM cell line in the cytoplasmic space, at a level significantly dependent on nanoparticles size and polydispesity index. Interestingly, by fluorescent studies, the authors demonstrated that nanoparticles were mostly distributed in the proximity of the perinuclear region after 12 and 24 h, without reaching the cell nucleus. This observation demonstrates the importance of the nanoparticles preparation method for improving their uptake. Interestingly, the internalization of these curcumin-loaded nanoparticles at micromolar concentrations was able to induce a persistent block in the G0/G1 phase of the cell cycle for up to 72 h, thus overwhelming the drug tolerance phenomenon, normally evidenced with free curcumin. The authors concluded by claiming that future in vitro cellular studies are necessary to shed light on the internalization and endocytosis mechanisms of the nanoparticles produced.

More recently, Masuelli et al. took advantage of different human and mouse MM cell lines (MM-B1, H-Meso-1, MM-F1 and #40a MM cells) to demonstrate that curcumin was able, in vivo, to inhibit MM cells survival in a dose- and time-dependent manner [66]. From a molecular point of view, curcumin in an earlier phase was able to trigger autophagic flux, but later on, this process was blocked and replaced by caspase-8 activation and subsequent apoptosis. Interestingly, this phenomenon of replacement of autophagy with apoptosis in cancer cells treated with chemotherapeutic agents has been extensively demonstrated [67,68]. Finally, in a mouse ortothopic model of MM, the intraperitoneal administration of curcumin increased the survival of the mice and reduced the risk of developing tumors [66].

Using a rat orthotopic model of sarcomatoid MM, Pouliquen et al. showed that intracavitary administration of curcumin was able to significantly decrease the tumor mass, inducing abundant necrosis [69]. The remaining neoplastic tissue displayed a reduction in the mitotic index and an increase in interleukin-6 and vimentin expression. Moreover, curcumin administered intraperitoneally induced an influx of macrophages in areas where a significant number of apoptotic cells was detected and produced an accumulation of CD8+ T cells at the periphery of remaining tumor tissue and an infiltration of normal tissues. Considering that this in vivo model of MM mimics the worst clinical conditions found in human patients affected by MM, the data produced could represent a good basis for evaluating the immune status modification induced by the treatment and to determine novel models of therapy for this malignancy.

Zhang et al., adopted the RN5 MM cell line to prove that curcumin was able to induce apoptosis via the mitochondrial pathway and caspase-independent and apoptosis-inducing factor-dependent pathways [70]. In detail, the authors demonstrated that curcumin induced apoptotic cell death of RN5 murine MM cell line via the AIF-dependent pathway and suppression of the PI3K-Akt-mTOR signaling pathway. The authors confirmed the data in an ectopic model of MM in mice, also demonstrating that curcumin inhibited angiogenesis in the tumor tissue.

**Table 1:** Laboratory studies examining the effects of curcumin in cancer.

First Author (Year) [ref]	Study Design	MM Cell Lines	Main Results
Yamauchi (2012) [63]	Laboratory study	ACC-MESO-1	curcumin was effective in reducing in dose-dependent manner cell viability through to autophagy
Miller (2012) [64]	Laboratory study	HMESO, H2595	growth-suppressive activity of H2461 curcumin on MM cells was through induction of pyroptosis and protection against inflammation
Serri (2017) [65]	Laboratory study	MSTO-211H	curcumin induced a persistent block in G0/G1 phase of the cell cycle up to 72 h, thus overwhelming the drug tolerance phenomenon
Masuelli (2017) [66]	Laboratory study	MM-B1, H-Meso-1, MM-F1 and #40a	curcumin inhibited cell survival in an earlier phase by triggering autophagic flux, but later on by activating apoptosis
Pouliquen (2017) [69]	Laboratory study	M5-T1	intracavitary administration of curcumin was able to significantly decrease the tumor mass in a rat orthotopic model of sarcomatoid MM
Zhang (2018) [70]	Laboratory study	RN5	curcumin was able to induce apoptosis via the mitochondrial pathway and caspase-independent and apoptosis-inducing factor dependent pathways
Di Meo (2019) [71]	Laboratory study	MSTO-221H, NCI-H2452	curcumin-C3complex^®^/Bioperine^®^ induced growth inhibition by apoptosis in all MM cell lines examined in a dose- and time-depended manner and reduced cell migration and cell invasive ability

Di Meo et al. took advantage of the curcumin-C3complex^®^/Bioperine^®^, a commercially standardized extract containing a ratio-defined mixture of three curcuminoids and piperine that greatly increased its bioavailability [71]. To note, several clinical trials have been started for investigating the effects of this formulation in human diseases, but to date, there have been no data on human MM [72]. Curcumin-C3complex^®^/Bioperine^®^ induced growth inhibition by apoptosis in all MM cell lines examined in a dose- and time-depended manner and reduced cell migration and cell invasive ability. The investigation of the molecular signaling pathway gave clues that the intrinsic apoptotic pathway is activated by this treatment. Finally, curcumin-C3complex^®^/Bioperine^®^ treatment of ectopic tumors in a MM xenograft mouse model in vivo was able to delay the growth of the tumors.

## 4. Conclusions

**Figure 2:**
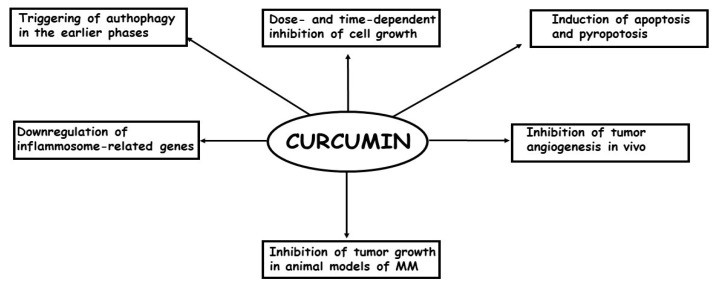
The most important in vitro and in vivo effects of curcumin in MM cells are depicted.

To date, few studies have examined the effects of curcumin treatment on MM cells both in vitro and in vivo [73]. Briefly, it has been demonstrated that: (a) curcumin is able to inhibit cell growth in a dose- and time-dependent manner; (b) the inhibition of cell growth is, at least, partially caused by an increase in apoptosis, mostly through the intrinsic pathways; (c) In the earlier phases of the treatment, curcumin causes an autophagic flux, but later, the process is blocked and replaced by apoptosis; (d) in some models, the most important phenomenon causing the inhibition of cell growth is pyroptosis; (e) curcumin treatment per os or intra-cavitary of in vivo models of MM is able to suppress the growth of the tumors; (f) curcumin treatment is able to enhance efficacy of the first-line anti-MM therapeutic cisplatin. In Figure 2, the in vitro and in vivo effects of curcumin on MM cells are summarized.

The data produced to date are promising, even if the relatively small number of scientific works produced, as well as the significant differences in experimental settings, do not allow us to reach definitive conclusions. In detail, the lack of a golden standard for the use of curcumin both in vitro and in vivo must be underlined and the fact that this represents a weak point when considering the potential use of this drug as an antineoplastic drug. Additional experimental work is necessary to better describe the molecular effects of curcumin on MM cells, to define the most suitable mixture of curcumin (complexed with nanoparticle, with piperine or with any other compound able to increase its bioavailability), and the most effective route of administration of the substance. Ongoing studies in our laboratory, for example, are investigating the possibility to increase the uptake in vivo of curcumin by pre-treating the tumor tissue by permeabilizing electric pulses, taking advantage of our expertise in electrochemotherapy [74,75,76]. This is a therapeutic strategy that takes advantage from the ability of electric pulses with peculiar characteristics, to greatly increase the permeability of the neoplastic cell membrane through modifications of the quaternary structure of the protein components of the membrane [74]. This application has been widely used in cancer therapy both in animals and in humans to increase the uptake of anti-cancer drugs by the neoplastic cells [76,77]. Hopefully, in the near future, it will be possible to include curcumin as a chemo-preventive agent or as a substance in combination with conventional chemotherapy in MM.

## Abbreviations

MM	Malignant mesothelioma
